# Loose Anagen Hair Syndrome

**DOI:** 10.4103/0974-7753.77513

**Published:** 2010

**Authors:** Rachita P Dhurat, Deepal J Deshpande

**Affiliations:** Department of Dermatology, TNMC and BYL Nair Charitable Hospital, Mumbai Central, Mumbai–400 008, India

**Keywords:** Histopathology, loose anagen hair, minoxidil, pathophysiology, trichogram

## Abstract

Loose anagen syndrome (LAS) is a benign, self-limiting condition where anagen hairs are easily and painlessly extracted. It is mainly reported in childhood; however, it may variably present in adulthood as well. The presence of anagen hair devoid of its sheath and with ‘floppy sock appearance’ is a characteristic feature of loose anagen hair (LAH) on trichogram. LAH can be seen in normal population and in alopecia areata. The percentage of LAH in LAS is more than 50%. The histopathological findings show clefting between the layers of hair and are very useful in differentiating LAS from alopecia areata. Here, a review on the diagnostic criteria and practical guidelines are discussed so as to enable the trichologist in managing this benign, self-limiting condition and differentiating it from the other causes of non-scarring alopecias.

## INTRODUCTION

The loose anagen hair (LAH), which is also known as loose anagen syndrome (LAS), is a disorder of abnormal anagen hair anchorage. The LAH is a sporadic or autosomal dominant disorder with variable expressivity that primarily affects children but occurrence in adults has also been reported.[1–10] It commonly occurs in the white population but it is not rare in dark-skinned individuals. Abdel-Raouf *et al*. Hamza *et al*.[11] reported a large collection of LAH syndrome in dark-skinned individuals.

## HISTORY

The syndrome was probably initially reported in literature in 1986 by Nodl and later by Zaun under the name of ‘syndrome of loosely attached hair in childhood’.[1] A few years later, the term LAS was coined by and first published in the American literature by Price and Gummer[2] and Hamm and Traupe[3] in 1989. All these reports describe the same essential clinical findings; the ability to gently and painlessly pluck anagen hairs that lack inner and outer root sheaths (ORSs). Lalevic-Vasic *et al*.[10] further described the histological features seen in this condition. In 1992, Baden *et al*.[5] elucidated ultrastructural abnormalities in this condition that led them to postulate possible mechanisms in this disorder. They also observed that there was an apparent hereditary component to this syndrome. There continues to be new reports of this condition, both in the United States as well as in France, Spain, Italy, the United Kingdom and Australia.

### Pathophysiology

The precise pathogenesis of LAS is not known; however, theories postulating an abnormality in the hair’s anchoring mechanism predominate. The inner root sheath (IRS) is thought to play an integral role in anchoring the hair shaft within the follicle and the first layer to keratinize playing a key role in the differentiation of the layers. Most authors believe that LAH results from a premature keratinization of the IRS that produces an impaired adhesion between the cuticle of the IRS and the cuticle of the hair shaft. However, Chapalain *et al*.[12] reported mutations in the gene encoding for the companion layer keratin (*K6HF*) in some members of the three of nine families with LAS. Their results suggest that the genetic basis of LAS may involve more than one gene encoding for keratins expressed in the IRS or in the companion layer (the innermost layer of the ORS). Another possible candidate may be the gene encoding for the new keratin (*K6IRS*) specific for the IRS.[13] This faulty keratinization leads to impaired adherence of the ORS–IRS, resulting in premature cessation of the anagen phase and account for reduced hair length. The findings of Chapalain indicate that the ORS–IRS adhesion is involved in at least some cases. This might explain why LAH is loosely anchored and the characteristic painless and easy pluckable. Measurement of the force required to pluck single scalp hairs in patients with LAS showed a mean value of 14 g, which is significantly lower than that of normal controls (48 g).[14] Price and Gummer attributed the short and sparse hairs in LAH syndrome to the short duration of anagen hair and the small size of the follicles.[2] The hair abnormalities in loose anagen syndrome are not constant and not all hair follicles are involved at a time. Chong *et al*.[15] studied the fluctuations in the number of hairs in the hair pull test and hypothesized an explanation for the variable positivity. Anagen is conventionally divided into six stages, anagen I–V (proanagen), when the hair shaft grows within the hair follicle, and anagen VI (metanagen), when the tip of the hair shaft emerges from the hair follicle. Anagen VI may have a number of substages associated with senescence and weakening of the adhesion between the IRS and hair cuticle layers, allowing easier extraction by the pull test. It is likely that anagen hairs in LAS only become ‘loose’ enough for extraction with a gentle hair pull at a specific point in the hair cycle. Hence, at different times, there will be different numbers of hairs at the specific stage in the hair cycle that allows them to be easily released. Therefore a negative hair pull test does not exclude LAS and a trichogram is essential to confirm the diagnosis.

### Clinical features

**Figure 1:**
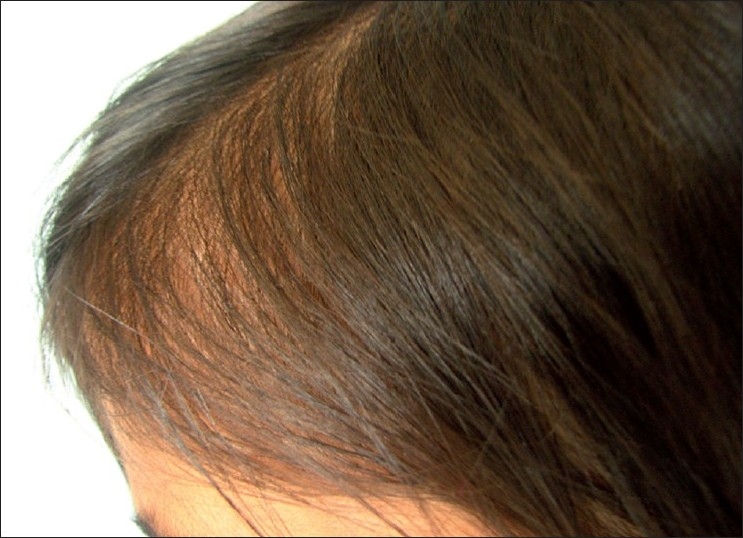
Patient with thin, sparse and unruly hair

Loose anagen syndrome is predominantly seen in young girls in between 2 and 6 years, but can be seen in boys as well. The condition is underdiagnosed in males because of hairstyle differences between boys and girls.[16] The typical complaint of the parent is that the child’s hair is lusterless and does not grow. Physical examination reveals sparse growth of thin, fine hair and diffuse or patchy alopecia [Figure 1]. Gentle traction results in hair that is painlessly removed; however, hair is not fragile. Hair may be of varying length and lusterless in appearance. Hair overlying the occiput tends to be rough or sticky and does not lie flat. The repeated rubbing of the occipital region of the head against the pillow at night pulls out more hair and this explains why the occiput is affected most.[11] The clinical presentation of LAS is heterogeneous. Presenting features are thin, sparse, fine hair or increased shedding of hair or patients whose hair is not growing or/and having odd texture: frizzy, unmanageable, unruly hair. Based on this, three phenotypes have been identified, with each having in common the finding of easily and painlessly extracted LAH on gentle hair pull. These phenotypes are:

1. Type A LAS, characterized by decreased hair density
2. Type B LAS, characterized by mainly unruly hair
3. Type C LAS, characterized by normal appearing hair with excessive shedding of LAH.

The above phenotypes appear to be age-dependent, with types A and B occurring in children, possibly evolving into the type C phenotype around the age of eight years and occurring in adults.[15]

LAS is usually restricted to scalp hair, but a patient with involvement of eyebrows and body hairs has been reported.[14]

### Associated conditions

Loose anagen hair syndrome is in most cases isolated, but it also occurs in association with hereditary or developmental disorders. These include coloboma, Noonan syndrome, hypohidrotic ectodermal dysplasia, EEC (ectrodactyly–ectodermal dysplasia–clefting) syndrome, trichorhinophalangeal syndrome, nail–patella syndrome, neurofibromatosis, trichotillomania, woolly hair.[17–26] and has been described with AIDS.[27] LAS can be a presenting feature in alopecia areata.[28,29]

## INVESTIGATIONS

### Hair pull test

The hallmark feature of LAS is the ability to extract anagen hairs easily and painlessly with a simple hair pull test. A tuft of 20–50 hairs is isolated and grasped close to the scalp between the thumb and index finger and gentle traction is then applied from the proximal to the distal end of hair. The traction exerted does not cause pain and is repeated from multiple sites on the scalp. However, presence of LAH at the pull test is not specific for LAS since it may also occur in healthy individuals. Diagnosis of LAS therefore relies on number and percentage of LAHs at the pull test and on trichogram. Olsen *et al*.,[30] state that the pull test results of children with LAS show more than three and often more than ten LAHs, compared with the pull test findings of normal children, which usually show one or two LAHs.

### Hair-pluck trichogram

**Figure 2:**
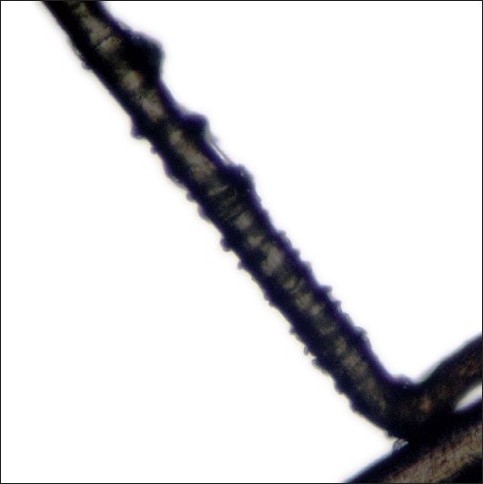
The characteristic ruffled appearance of the cuticle is referred as the ‘Floppy sock appearance’

**Figure 3:**
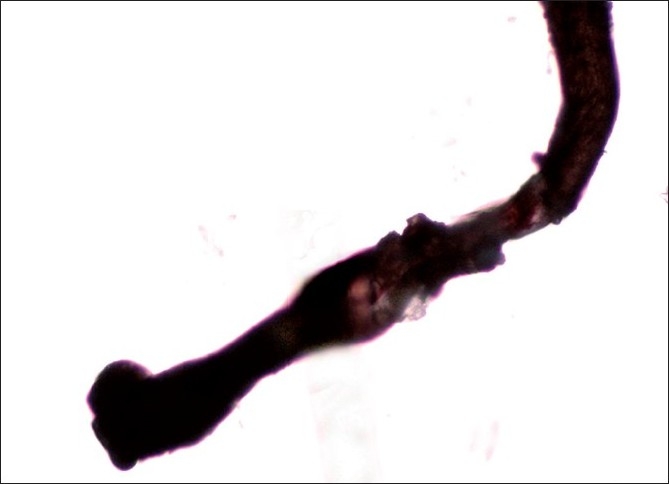
Trichogram showing misshapen anagen bulb and long, twisted hair

**Figure 4:**
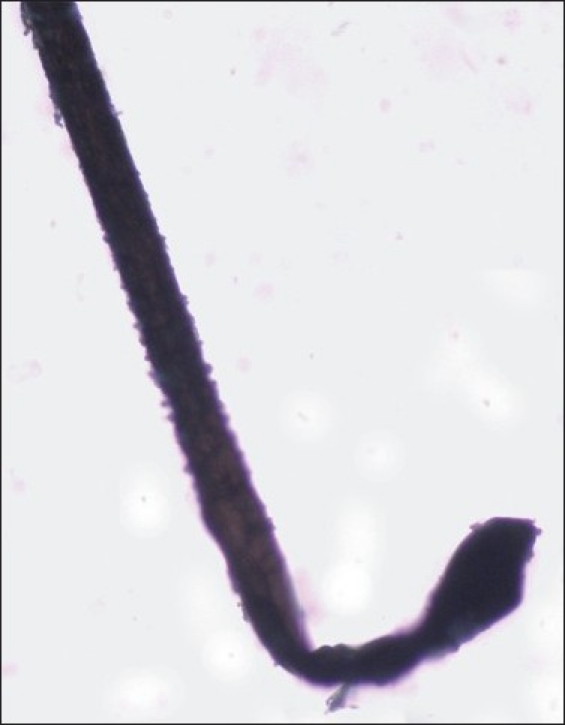
The hair bulb is at an acute angle to the shaft resembling a ‘mouse- tail’

A tuft of around 20–40 hairs are isolated and a pair of epilating forceps with rubber tubing over the tips are applied near the scalp. The epilating forceps are then rotated 90° so that the hairs do not slip through the teeth. A smooth forceful pull is then applied, rapidly extracting the hairs for examination under the microscope. Staining with cinnamaldehyde which selectively stains the citrulline rich IRS is helpful.[4] The trichogram reveals predominance of anagen hairs (≥70%) devoid of the outer and IRSs. The proximal segment of hair shaft closest to the root appears distorted and twisted with the characteristic ruffling of the cuticle seen. This has been described as the ‘floppy sock appearance’, which is a characteristic of LAS [Figure 2]. Other findings reported are misshapen anagen bulbs and long, tapered and twisted hairs [Figure 3]. Some of the hair bulbs are positioned at an acute angle to the shaft resembling mouse tails[11,29] [Figure 4]. A few or no telogen hairs are present. Among 37 patients with LAS, trichorrhexis nodosa was seen in two and pseudotrichothiodystrophy, a hair shaft disorder characterized by irregular alternating dark and light bands with twisted shafts under polarized microscopy was seen in three in addition to typical LAH.[17]

### Histopathology

**Figure 5:**
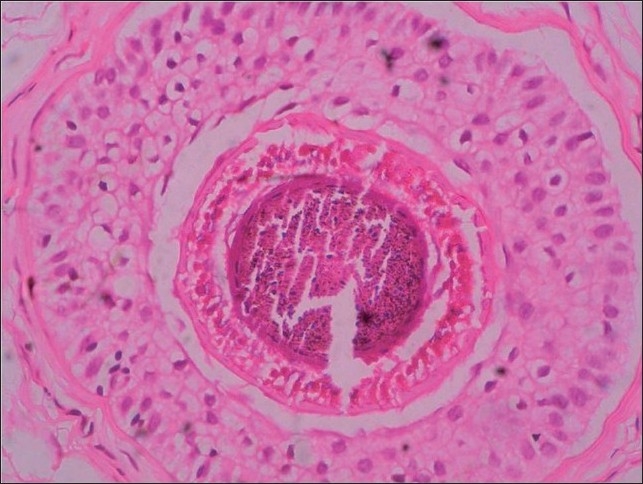
On ×40, histopathology shows clefting within hair shaft, inner and outer root sheaths

On histopathology, the characteristic findings are clefting between IRS, IRS and ORS, ORS and fibrous sheath and lack of inflammation. The clefting is considered to be an artifact while processing and can be found in normal scalp biopsies but in LAS this process is prominent and exaggerated.[31] We found degenerative changes with abnormal cornification of IRS and clefting within the IRS, between the non-cornified cortex and IRS, between the IRS and ORS in our patient of LAS [Figure 5]. In our experience, the hair pull test severity may correlate with the multiple clefting seen on histopathology. Reports of patients with diffuse hair loss with clinical and trichogram findings suggestive of LAH, however, were found to have peribulbar lymphocytic infiltrate suggestive of alopecia areata on scalp biopsy.[28,29] Therefore we recommend a scalp biopsy in every patient suspected to have LAH syndrome.

### Electron microscopy

The epilated anagen hair shows rippling of the hair cuticle of the proximal hair shaft. Triangular or flattened shapes and longitudinal grooves and torsions are also occasionally present. The only characteristic finding is a premature keratinization of the cells of the Henle and Huxley layers of the IRS.[32] Such premature keratinization could interfere with the normal interdigitation of the IRS cuticle and the hair shaft cuticle and disturb anchorage of anagen hairs.[11]

### Indirect immunofluorescence

Indirect immunofluorescence findings revealed the positive staining in the outer root sheath (ORS) reacted with *K6HF* antibodies.[11]

## DIAGNOSTIC CRITERIA

The presence of typical LAH in the trichogram of some normal individuals led some investigators to state that light microscopic findings in LAH syndrome are not pathognomonic. Diagnosis of LAH syndrome therefore relies on the number and percentage of LAH at the hair pull test and on trichogram. Tosti *et al*.,[6] proposed diagnostic criteria for LAS on the basis of hair pull test and trichogram:

1. Positive pull test results with painless extraction of at least 10 LAHs.
2. The presence of more than 80% LAH on trichogram.

However, these criteria are too strict and only permit the severely affected to be diagnosed. Therefore, Tosti *et al*.[32] revised these criteria and suggested that the diagnosis of LAS should not be made if the trichogram does not show at least 70% LAH. Cantatore-Francis *et al*.[17] proposed that LAH syndrome should only be diagnosed when there is more than 50% of LAHs on a trichogram.

## DIFFERENTIAL DIAGNOSIS

Alopecia areata is the most important differential diagnosis. In LAH syndrome, however, the patches are not sharply demarcated and are not totally devoid of hair, and nail changes are lacking. The trichogram would show an increase in dystrophic and/or telogen hairs in the margins of progressive patches of alopecia areata.[11]

Trichotillomania and LAH syndrome share striking predominance of anagen hair, however, trichogram shows preserved root sheath in trichotillomania. In telogen effluvium, the pull test revealed increase telogen hairs (>50%). Anagen effluvium is seen frequently following administration of cancer chemotherapeutic agents and the extracted hairs are dystrophic with tapered proximal ends.

## TREATMENT AND PROGNOSIS

Most cases of LAS resolve spontaneously with age and this could be contributed to androgen that may affect the follicle indirectly through a mesenchymal–epithelial interaction.[11] Therefore some hair acquire normal anagen appearance. However some patients continue to show a small percentage of LAH at the pull test or on trichogram indicating that the defect of hair shaft anchorage is still present even if to a lesser degree.[32] The successful use of minoxidil is encouraging and may be a reasonable first-line therapy for patients with disease at the severe end of the LAH syndrome spectrum.[17] The exact mechanism by which it works is not known. It may prolong the anagen phase and stimulate growth of hair follicles.

## CONCLUSIONS

Loose anagen hair syndrome may sometimes be confused with other hair disorders, but the history, clinical findings and light microscopy of pulled hair should easily exclude other diagnosis. To summarize, some important practical points to remember while treating a child complaining of hair loss are:

1. A high index of suspicion in a child younger than six years
2. A hair pull test and trichogram findings are essential for the diagnosis
3. Spontaneous resolution in most cases, but minoxidil may fasten resolution and may be used in severe cases.
